# West Nile Virus Associations in Wild Mammals: An Update

**DOI:** 10.3390/v11050459

**Published:** 2019-05-21

**Authors:** J. Jeffrey Root, Angela M. Bosco-Lauth

**Affiliations:** 1 U.S. Department of Agriculture, National Wildlife Research Center, Fort Collins, CO 80521, USA; 2 Department of Biomedical Sciences, College of Veterinary Medicine and Biomedical Sciences, Colorado State University, Fort Collins, CO 80523, USA; mopargal@colostate.edu

**Keywords:** antibodies, artiodactyla, carnivore, experimental infection, exposure, flavivirus, mammal, mesocarnivore, rodent, West Nile virus, wildlife, viremia

## Abstract

Although West Nile virus (WNV) is generally thought to circulate among mosquitoes and birds, several historic and recent works providing evidence of WNV activity in wild mammals have been published. Indeed, a previous review tabulated evidence of WNV exposure in at least 100 mammalian species. Herein, we provide an update on WNV activity in wild and select other mammals that have been reported since the last major review article on this subject was published in early 2013. Of interest, new species, such as Hoffman’s two-toed sloths (*Choloepus hoffmanni*), are now included in the growing list of wild mammals that have been naturally exposed to WNV. Furthermore, new instances of WNV viremia as well as severe disease presumably caused by this virus have been reported in wild mammals (e.g., the Virginia opossum [*Didelphis virginiana*]) from natural and semi-captive (e.g., zoological institution) settings. Regrettably, few recent challenge studies have been conducted on wild mammals, which would provide key information as to their potential role(s) in WNV cycles. Largely based on these recent findings, important future lines of research are recommended to assess which mammalian species are commonly exposed to WNV, which mammal species develop viremias sufficient for infecting mosquitoes, and which mammal species might be negatively affected by WNV infection at the species or population level.

## 1. Introduction

The global distribution of West Nile virus (WNV) has greatly expanded over the last two decades following its introduction into the New World during 1999. While the cycles of WNV primarily involve mosquitoes and birds [1], previous work has noted that a diversity of wild mammal species have been naturally exposed to WNV [2]. Recent evidence suggests that these trends continue. Although limited in terms of the number of species tested, select wild mammal species produce viremias of titers at which they would be considered moderately competent hosts that likely have the capacity to infect mosquitoes [2]. For example, multiple studies associated with fox squirrels (*Sciurus niger*) have reported that this species can develop viremia of 10^5^ pfu/mL or greater [3,4,5]. This indicates these species could act as reservoir hosts for the virus, thereby potentially exposing more humans and animals to WNV. However, no wild mammals that have been evaluated to date develop viremias to levels that have been observed from highly competent bird species (avian competence levels reviewed by [6]).

The potential role of wild mammal involvement in WNV cycles has been postulated for well over a decade. Concern about the potential significance of these species has not waned, as during recent years it has been considered important to assess the possible role of peridomestic mammals in WNV cycles should exotic strains of the virus be introduced into certain regions [7]. Notably, among the 30 flaviviruses evaluated, modeling efforts indicated that WNV has the highest host species diversity within this genus, with 194 known bird and mammal hosts (based on certain criteria) and many more hosts predicted by macro-ecological modeling [8]. The objective of this review is to summarize recent WNV activity in wild mammals (and select other mammals not typically summarized in the literature) that has been reported since the last major review article on this subject that was published during 2013 [2]. In addition, several potential key priorities of WNV research in wild mammals are discussed.

## 2. West Nile Virus (WNV) Exposures in Wild Mammals

Several wild mammal exposures to WNV have been reported in recent years. While some are consistent with those species that have been reported previously, others represent species with no previous documented exposures. The majority of recent exposures have been reported in artiodactyls, carnivores and mesocarnivores, rodents, and non-human primates (Table 1, Table 2, Table 3 and Table 4). In general, evidence of these exposures has been ascertained through detections of antibodies. However, some new reports of viremia in wild-caught mammals as well as apparent severe disease in some species have been documented. While the majority of the studies discussed in this review utilized multiple flaviviruses to determine the WNV sero-status of the animals that were studied, some did not. Therefore, this limitation should be considered in the interpretation of the serology presented in these instances (see footnotes in Table 1 and Table 2). Summaries of these recent WNV exposures are listed below.

### 2.1. WNV in Artiodactyls

Evidence of exposure to WNV has been previously detected in a diverse group of artiodactyls (cloven-hooved mammals), with many detections associated with various deer species [9,10,11]. Similar to earlier reports [2], more WNV surveys of artiodactyls have been reported from the Old World in recent years (Table 1). For example, WNV neutralizing antibodies were recently reported in wild boars (*Sus scrofa*) and roe deer (*Capreolus capreolus*) in Serbia [12]. Similarly, an ambitious (*n* = 1023) WNV serosurvey conducted on artiodactyls over a 19-year period in southern Moravia (Czech Republic) was recently published [13]. Overall, WNV antibodies were identified in 5.2% of the artiodactyls tested, including 4.8% of 105 roe deer, 4.1% of 148 red deer (*Cervus elaphus*), 6.3% of 287 fallow deer (*Dama dama*), 9.9% of 71 mouflons (*Ovis* sp.), and 4.1% of 412 wild boars [13].

Sixty-nine of 545 wild boar from Spain had antibodies to flaviviruses, but hemolysis limited the number of samples that could be tested by micro virus-neutralization tests (micro VNT) [14]. Only 21 of the 69 samples positive for flaviviruses were further evaluated, of which nine were positive for WNV neutralizing antibodies with titers ranging from 1:10 to 1:160 [14]. In a second study from Spain, evaluating the antibody prevalence of WNV and antigenically related flaviviruses, serology was conducted on thousands of wild ruminant samples (red deer, fallow deer, mouflon, and roe deer), and low antibody prevalence was noted, ranging from 3.4% for red deer to 0% for roe deer [16]. After accounting for possible coinfections, the overall WNV antibody prevalence was estimated to be ≤2.4% [16]. A third study conducted in Spain was based largely on red deer and wild boar. The overall WNV/flavivirus (the methods used could not definitively distinguish between the two categories) seroprevalence was estimated at approximately 4.04% for wild boar and 0.23% for red deer that were collected in multiple bioregions [15]. In addition, three of 100 privately-owned dromedary camels (*Camelus dromedarius*) were assessed to be antibody positive (by enzyme-linked immunosorbent assay [ELISA]) for WNV in the Canary Islands, Spain [17]. The low number of antibody positive animals led the authors to conclude that WNV was not currently circulating among and within the two herds tested [17]. Although WNV antibodies in camels have been reported for some time, the first WNV isolation was recently reported from a dromedary camel calf from the United Arab Emirates [18].

A serosurvey of antibodies to alphaviruses and flaviviruses in wild mammals in the greater Congo basin was recently reported [20]. Among the artiodactyls tested, a single African forest buffalo (*Syncerus caffer nanus*) from Garamba National Park in the Democratic Republic of Congo was assessed to be antibody positive [20].

Of 1,508 white-tailed deer (*Odocoileus virginianus*) serum samples tested, an overall WNV seroprevalence of 6.0 % was detected from 18 U.S. states and the U.S. Virgin Islands [21]. Among the 19 collection locations, at some of which no antibodies were detected, Louisiana yielded the highest seroprevalence at 18.7% [21]. Ten percent of 29 serum samples from privately owned camels submitted to an Animal Health Diagnostic Laboratory in New York, USA, were WNV antibody positive by ELISA [19]. Although testing was conducted on a captive herd, recently published filter paper studies of WNV serology (by ELISA) in reindeer (*Rangifer tarandus tarandus*) suggest that reindeer are exposed to this virus in Alberta, Canada and that user-friendly filter paper methods appeared to work well to collect samples for subsequent antibody testing [22].

### 2.2. WNV in Non-Human Primates

Previous published accounts of wild non-human primate exposure to WNV have been primarily associated with the Old World, especially Madagascar [23,24,25]. A recent study greatly expanded the geographic distribution of known WNV exposures in wild non-human primates, as a >8% antibody prevalence (based on monotypic responses) was noted in free-ranging black howlers (*Alouatta caraya*) in northeastern Argentina [26]. A small number of mountain gorillas (*Gorilla beringei beringei*), one of which was generally thought to be transient between the Democratic Republic of the Congo and Rwanda, were recently reported to have antibodies reactive with WNV [20].

Two primate species, rhesus macaques (*Macaca mulatta*) and common marmosets (*Callithrix jacchus*), were recently experimentally infected with a European strain (WNV-Ita09) of WNV [27]. Both species exhibited productive infections, exhibited a viremic period of multiple days, and produced WNV RNA-positive tissues [27]. Of interest, common marmosets developed a higher peak and longer-lasting viremia and also had a broader tissue distribution that was positive for viral RNA [27]. Neither species in this study developed clinical disease. 

### 2.3. WNV in Carnivores and Mesocarnivores

Even though it is a marsupial, the Virginia opossum (*Didelphis virginiana*) is considered to be a mesocarnivore in the U.S. While multiple WNV antibody detections in Virginia opossums were reported well over a decade ago [28,29,30], some important information associated with this species has been recently published (Table 2). First, a viremic Virginia opossum was detected in northwestern Missouri, USA during the summer of 2012 [31]. At the time this wild-caught animal was sampled, it had a titer of 10^2.5^ pfu/mL [31]. Because this was a sample from a wild-caught animal, the exposure period of the animal is unknown. Therefore, the titer listed above may or may not represent the peak viremia titer from this species. Second, although antibodies have been detected in many individuals, thereby suggesting that they are often resistant to severe disease, WNV may have the ability to cause lethal disease in the Virginia opossum in some instances. For example, a recent publication was suggestive of a fatal WNV infection concurrent with pulmonary lepidic-predominant adenocarcinoma in a Virginia opossum [32]. The WNV diagnosis was based upon viral RNA from pooled tissues and histologic lesions that were observed in multiple organs [32]. Considering the new information on this species, the Virginia opossum is a logical choice for laboratory-based evaluations of its peak viremia and disease dynamics following deliberate infections with WNV.

Evidence of WNV exposure has been long-documented in other mesocarnivores. Raccoons (*Procyon lotor*) and striped skunks (*Mephitis mephitis*) have both exhibited relatively high seroprevalence rates in some locations during earlier studies [28,29,36]. A recently published paper identified very high (>50%) antibody prevalence in raccoons sampled on Long Island, New York, USA, but indicated that the role of raccoons in WNV epidemiology cannot be elucidated because of a lack of studies evaluating viremia in this species [19]. However, a study of this type was published in 2010 and indicated that raccoons can rarely develop viremias approaching 10^5^ pfu/mL, thereby suggesting they are unlikely to be important amplifying hosts of this arbovirus [37]. A second study, conducted in Ontario, Canada, noted a low WNV seroprevalence in raccoons (4%) and a moderate seroprevalence in striped skunks (17%) [33].

WNV exposures in multiple captive and wild bear species (*Ursus* spp.) have been noted in historical literature, and have been occasionally associated with severe disease [38,39,40]. Similarly to the low prevalence that was previously reported in New Jersey [39], a recent study reported a low WNV antibody prevalence in black bears (*U*. *americanus*) from Maryland, USA [34]. Furthermore, one of 24 wild Eurasian brown bears (*U. arctos arctos*) sampled from six regions of Slovakia was antibody positive for WNV in the Nízke Tatry region of this country [35].

Antibodies to flaviviruses were detected in 21 of 103 red foxes (*Vulpes vulpes*) in Spain [14]. Due to the degree of hemolysis of serum samples, only one individual was tested by micro VNT; this individual was confirmed positive for WNV antibodies [14]. In the same study, one of 6 stone martens (*Martes foina*) was positive for antibodies to flaviviruses, but the single ELISA positive individual was not confirmed positive for WNV neutralizing antibodies [14].

### 2.4. WNV in Rodents

Several WNV investigations have been associated with tree squirrels (e.g., *Sciurus* spp. and *Tamiasciurus* spp.). This is likely due to the fact that previous work has indicated that some tree squirrel species (e.g., fox squirrel and eastern gray squirrel [*S. carolinensis*]) are commonly exposed to WNV [29,30,41] and at least two species can develop viremia profiles that may be sufficient to infect mosquitoes [3,4,5,42]. During a recent survey in Georgia, USA, a relatively high antibody prevalence (36%) was noted in eastern gray squirrels, but none of the 69 animals tested showed evidence of viremia at their time of capture [43]. It was also postulated that season had an effect on mosquito infection status and eastern gray squirrel seroprevalence, as both peaked during the summer [43]. Mortality in WNV-positive fox squirrels was recently noted in Michigan, USA [44]. Two of the tested individuals had pooled tissue samples that were WNV positive by reverse transcriptase polymerase chain reaction (RT-PCR), but the authors questioned the significance of this finding due to the lack of immunoreactivity of these tissues and the presence of other pathogens in the animals [44]. An additional WNV survey in tree squirrels was recently conducted in Europe (Table 3). A relatively small percentage of introduced eastern gray squirrels were assessed to have antibodies generically reactive with flaviviruses in Italy, but only one individual was assessed to be antibody positive for WNV [45]. 

Groundhogs (*Marmota monax*) have been previously assessed for WNV exposure in the U.S. In an earlier serosurvey conducted in part in the eastern U.S., none of the two groundhogs tested in Ohio were positive for antibodies specific to WNV [29], but one of three individuals tested positive for antibodies in Maryland [30]. A recent study conducted in Ontario, Canada, reported that three groundhogs (17.6% of total tested) had WNV-neutralizing antibodies [33].

Commensal rodents of the genera *Rattus* and *Mus* have been previously assessed for WNV exposure, both of which have yielded evidence of antibody-positive individuals [29,48]. A recent study evaluated two species from these genera in highly peridomestic settings in Merida, Mexico. While many black rats (*R*. *rattus*) and house mice (*M*. *musculus*) had antibodies reactive with flaviviruses, none of the individuals positive for WNV antibodies by plaque reduction neutralization tests (PRNT) could be confirmed as definitively WNV due to low or similar PRNT_90_ titers with other flaviviruses [49]. However, a single black rat had a WNV titer of 1:20 and did not react with the six other viruses tested [49].

Of the 90 sera tested from four rodent species (yellow-necked field mouse [*Apodemus flavicollis*], wood mouse [*A*. *sylvaticus*], bank vole [*Myodes glareolus*], and garden dormouse [*Eliomys quercinus*]) collected in Italy, a total of four yellow-necked field mice sera tested positive for WNV antibodies by ELISA; two of the four samples were confirmed as WNV (at low titers) by virus neutralization tests [46]. In addition, it has been reported that WNV has been sporadically isolated from yellow-necked field mice and bank voles [47].

### 2.5. WNV in Other Wild Mammals

Recently, WNV antibodies were reported during multiple years in free-ranging Hoffman’s two-toed sloths (*Choloepus hoffmanni*) at annual prevalences of 8% and 27% during the mid-2000s [50]. However, during the same study, which was conducted in Costa Rica, the virus was not detected in brown-throated sloths (*Bradypus variegatus*) [50]. To our knowledge, this is the first report of documented WNV exposures in sloths (Table 4).

While several bats from the Yucatan Peninsula of Mexico were shown to have antibodies reactive with generic flaviviruses, none were shown conclusively to have WNV antibodies, even though eight individuals had PRNT_90_ titers ranging from 1:20–1:40 [52]. Chiropteran exposures to WNV, however, have been commonly reported during earlier observations in the Old and New Worlds [2]. More recently, WNV exposure (neutralizing antibodies) was reported in a small number of African straw-colored fruit bats (*Eidolon helvum*) and little epauletted fruit bats (*Epomophorus labiatus*) that were captured in Uganda [51]. Among eight total species tested, only five of 626 individuals were confirmed as having WNV neutralizing antibodies [51]. 

Documented WNV exposures in elephants have historically been largely restricted to captive populations. For example, multiple Asian elephants (*Elephas maximus*) associated with the Bronx Zoo/Wildlife Conservation Park as well as captive populations (presumably Asian elephants) in Florida were assessed to be antibody positive following the 1999 introduction of WNV into the New World [53,54]. Of interest, a more recently published study reported that 100% (*n* = 31) of African elephants (*Loxodonta africana*) from the greater Congo basin had neutralizing antibodies against WNV, thereby indicating widespread exposure in this wild population [20].

### 2.6. Select Surveys that Failed to Detect WNV Exposure in Wild Mammals

While many studies have documented wild mammal WNV exposures during recent years, some have not detected exposures during targeted surveys. For example, a survey that included more than 100 mammals (primarily rodents) did not report any exposure among the 12 species (some captive) tested in Trinidad [55]. Similarly, an arbovirus wildlife survey of rodents (n = 14) and bats (n = 146) collected in southern Mexico did not yield molecular evidence of WNV infection [56]. WNV antibodies were absent in 70 water buffalo (*Bubalus bubalis*; presumably farmed) sampled in Turkey [57]. None of the serum samples from 49 wild Geoffroy’s spider monkeys (*Ateles geoffroyi*) and four black howler monkeys (*Alouatta pigra*) sampled in Mexico neutralized WNV [58]. Similarly, none of 49 wild and captive black-striped capuchins (*Sapajus libidinosus*) and blond capuchins (*S. flavius*) were antibody positive for WNV in Brazil [59].

Although serum samples collected from several wild mammal species in Ontario, Canada were positive for WNV antibodies, 0/22 samples from eastern gray squirrels and 0/1 samples from a red squirrel (*Tamiasciurus hudsonicus*) were positive [33]. Considering that there was documented WNV activity in other wildlife species in this area during the same time-frame [33] and high seroprevalence rates have been observed in these tree squirrel species previously (often much higher than other mammals sampled in the same regions) [29,30], this result is surprising, but may be influenced by the presumably longer life-spans of the majority of the other mammal species tested. 

## 3. Discussion

Over the past several decades, human population growth and land use has led to increasingly common interactions with wildlife. Thus, understanding the role wild animals play in the epidemiology of zoonoses is critical in order mitigate the spread of disease to and from hosts of zoonotic pathogens. There are several questions associated with WNV in wild mammals that, if addressed, would help to elucidate their potential roles in WNV epidemiology. First, targeted serosurveys of wild mammals are still lacking from some regions. Indeed, during recent years, the need for comprehensive mammalian WNV serosurveys to assess potential mammalian hosts in some regions has been proposed [60]. Aside from examining the possible mammalian involvement in WNV cycles, serosurveys of wild mammals can also provide a valuable and highly localized surveillance mechanism for WNV if key species are targeted. For example, monitoring tree squirrels for WNV has been proposed as a useful surveillance tool for WNV [29], especially when coupled with mark-recapture surveys [41]. Wild mammals could in this manner serve as sentinel species for regions with high outbreak potential.

Second, more experimental infections need to be conducted to assess if any mammalian species have the capacity to replicate higher levels of virus than those that have been previously assessed and to evaluate clinical disease. Unfortunately, very few new experimental infection studies associated with WNV viremia in wild mammals have been conducted during the past decade. Logical species to target for these types of studies include those that are known to have been commonly exposed to WNV but for which data on viremia is lacking. For example, while a field study indicated that the Virginia opossum can develop viremia following WNV infection [31], whether or not this species can produce viremia levels sufficient for infecting mosquitoes remains unknown. Furthermore, due to their potential involvement in urban cycles, several peridomestic mammals, which often live within close proximity to humans, are an obvious choice. Considering the recently published WNV viremia and disease in Virginia opossums detected in the field and from a zoological institution [31,32], this species represents a clear choice for further evaluation in controlled settings. In addition, some mammalian species are already known to have the capacity for moderate levels of viremia; therefore, ecologically similar species in different regions (e.g., Old World tree squirrels) represent an obvious line of useful research on this subject [2].

Third, aside from public health implications, WNV could have impacts on mammalian species or populations of conservation concern. In view of the severity of WNV disease in other incidental mammalian hosts, including humans and horses, it is logical to assume that there could be mammalian species equivalently impacted by this disease. For example, WNV is known to have the capacity to cause morbidity or mortality in multiple wild mammal species, including Virginia opossums and tree squirrels [32,61]. Thus, considering the lack of base-line information on WNV exposure rates and severity of disease caused by the virus for many mammalian species, surveillance of key species and populations of conservation concern could prove valuable for the management of these types of populations. Targeted surveillance and seroprevalence studies can then be used to guide experimental infection studies to allow researchers to investigate whether or not the presence of WNV is a source of concern for the conservation of key wildlife species. Previous studies involving bird species whose populations are impacted by WNV, including ruffed grouse (*Bonasa umbellus)*, indicate that the vaccination of wildlife can provide a valuable tool for protecting these animals [62]. Similarly, should a wild mammalian species whose population is heavily-impacted by WNV be determined, vaccination efforts could then be directed at those animals if an appropriate vaccine were available. 

## Figures and Tables

**Table 1:** Recently reported natural exposures of artiodactyls to West Nile virus.

Common Name	Scientific Name	Detection Type	Location	Reference
Wild boar	*Sus scrofa*	Antibodies	Serbia	[12]
		Antibodies	Czech Republic	[13]
		Antibodies^a^	Spain	[14]
		Antibodies^a^	Spain	[15]
Roe deer	*Capreolus capreolus*	Antibodies	Serbia	[12]
		Antibodies	Czech Republic	[13]
Red deer	*Cervus elaphus*	Antibodies	Czech Republic	[13]
		Antibodies^b^	Spain	[16]
		Antibodies^a^	Spain	[15]
Fallow deer	*Dama dama*	Antibodies	Czech Republic	[13]
		Antibodies^b^	Spain	[16]
Mouflon	*Ovis* sp.	Antibodies	Czech Republic	[13]
		Antibodies^b^	Spain	[16]
Dromedary camel	*Camelus dromedarius*	Antibodies^a,c^	Spain	[17]
		Virus	UAE^d^	[18]
“Camel”	Not listed^e^	Antibodies^a,c^	USA^f^	[19]
African forest buffalo	*Syncerus caffer nanus*	Antibodies	DRC^g^	[20]
White-tailed deer	*Odocoileus virginianus*	Antibodies	Multiple USA	[21]
Reindeer	*Rangifer tarandus tarandus*	Antibodies^a,c^	Alberta, CA	[22]

^a^ Indicates that a single test (e.g., enzyme-linked immunosorbent assay [ELISA]) was used, samples were not tested against multiple flaviviruses, or it is unclear if samples were tested against multiple flaviviruses. Therefore, all detections may or may not represent WNV. ^b^ Data were presented as WNV and antigenically related flaviviruses. ^c^ Animals were from a privately owned collection. ^d^ United Arab Emirates. Original paper did not list specific location of animal and if animal was privately owned of feral. ^e^ Species of camel was not listed in original paper. Animals were privately owned. ^f^ Serum samples were sent to a diagnostic laboratory. The actual locations of where the privately owned animals were sampled was not listed in the original paper. ^g^ Democratic Republic of the Congo.

**Table 2:** Recently reported natural exposures of carnivores and mesocarnivores to West Nile virus.

Common Name	Scientific Name	Detection Type	Location	Reference
Virginia opossum^a^	*Didelphis virginiana*	Virus	MO, USA	[31]
		Viral RNA/histopathologic	Quebec, CA	[32]
Raccoon	*Procyon lotor*	Antibodies^b^	NY, USA	[19]
		Antibodies	Ontario, CA	[33]
Striped skunk	*Mephitis mephitis*	Antibodies	Ontario, CA	[33]
Black bear	*Ursus americanus*	Antibodies	MD, USA	[34]
Eurasian brown bear	*Ursus arctos arctos*	Antibodies	Slovakia	[35]
Red fox	*Vulpes vulpes*	Antibodies^b^	Spain	[14]

^a^ Not a member of the mammalian order Carnivora but is considered to be a North American mesocarnivore. ^b^ Indicates that a single test (e.g., ELISA) was used, samples were not tested against multiple flaviviruses, or it is unclear if samples were tested against multiple flaviviruses. Therefore, all detections may or may not represent WNV.

**Table 3:** Recently reported natural exposures of rodents to West Nile virus.

Common Name	Scientific Name	Detection Type	Location	Reference
Fox Squirrel	*Sciurus niger*	Viral RNA	MI, USA	[44]
Eastern gray squirrel	*Sciurus carolinensis*	Antibodies	Italy^a^	[45]
		Antibodies	GA, USA	[43]
Groundhog	*Marmota monax*	Antibodies	Ontario, CA	[33]
Yellow-necked field mouse	*Apodemus flavicollis*	Antibodies	Italy	[46]
		Virus	Europe^b^	[47]
Bank vole	*Myodes glareolus* ^c^	Virus	Europe^b^	[47]

^a^ Species is introduced into Italy. ^b^ Reference did not give a specific location. ^c^ Listed in original paper as *Clethrionomys glareolus*.

**Table 4:** Recently reported natural exposures of non-human primates and other wild mammals to West Nile virus.

Common Name	Scientific Name	Detection Type	Location	Reference
Black howler	*Alouatta caraya*	Antibodies	Argentina	[26]
Mountain gorilla	*Gorilla beringei beringei*	Antibodies	DRC^a^/Rwanda	[20]
Hoffman’s two-toed sloth	*Choloepus hoffmanni*	Antibodies	Costa Rica	[50]
African straw-colored fruit bat	*Eidolon helvum*	Antibodies	Uganda	[51]
Little epauletted fruit bat	*Epomophorus labiatus*	Antibodies	Uganda	[51]
African elephant	*Loxodonta africana*	Antibodies	DRC^a^	[20]

^a^ Democratic Republic of the Congo.
